# Editorial: Adaptive immune resistance in cancer therapy

**DOI:** 10.3389/fphar.2023.1277902

**Published:** 2023-08-25

**Authors:** Aimin Jiang, Ouyang Chen, Zhigang Liu, Hongzhou Cai, Linhui Wang, Lin Qi

**Affiliations:** ^1^ Department of Urology, Changhai Hospital, Naval Medical University, Shanghai, China; ^2^ Department of Cell Biology, Duke University Medical Center, Durham, NC, United States; ^3^ Dongguan Key Laboratory of Precision Diagnosis and Treatment for Tumors, Dongguan, Guangdong, China; ^4^ Department of Urology, Jiangsu Cancer Hospital and The Affiliated Cancer Hospital of Nanjing Medical University and Jiangsu Institute of Cancer Research, Nanjing, Jiangsu, China; ^5^ Department of Orthopedics, The Second Xiangya Hospital, Central South University, Changsha, Hunan, China; ^6^ Hunan Key Laboratory of Tumor Models and Individualized Medicine, The Second Xiangya Hospital, Changsha, Hunan, China

**Keywords:** adaptive immune resistance, tumour microenvironment (TME), immunotherapy, biomarker, prognosis

Tumours employ various strategies to adapt and ultimately evade the immune system’s attack, collectively known as the adaptive immune resistance (AIR) (Morad et al., 2021). The initial AIR mechanism to be defined and validated therapeutically is the targeted induction of programmed cell death 1 ligand 1 (PDL1) by interferon-γ within the tumour. The use of antibodies to block the binding of PDL1 to its receptor, PD1 (referred to as anti-PD therapy), has led to remission in a notable subset of patients with advanced-stage cancer, particularly in solid tumours (Vasan et al., 2019; Sharma et al., 2021). However, several clinical trials investigating the combination of anti-PD therapy with other anti-tumour drugs, without a strong mechanistic rationale, have failed to identify a synergistic or additive effect (Hegde and Chen, 2020). Given the aforementioned concerns, it is imperative to identify specific AIR mechanisms that hold significant importance in the development of novel drugs and the enhancement of cancer treatment efficacy.

To address this pressing issue, a comprehensive analysis of AIR is conducted through an examination of 3 literature reviews and 11 original research papers authored by a total of 92 individuals. These collective studies bring forth valuable insights into the mechanisms and interconnectedness between AIR and the tumour microenvironment (TME).

Inhibitors targeting PD-1 and PD-L1 have become extensively utilized in the treatment of various types of cancer. To gain a comprehensive understanding of the current research landscape and pinpoint promising areas of investigation in the realm of PD-1/PD-L1 inhibitors, Lai et al. conducted an in-depth analysis utilizing bibliometric and visualized techniques. Their findings revealed that the United States is leading the country in this field, contributing a significant 42.03% of the total publications. Notably, the journal “Value in Health” emerged as the most productive journal in terms of publications. Moreover, the journal “New England Journal of Medicine” played a prominent role in the research network. The study also sheds light on active collaborations between countries and research institutes, highlighting the collaborative nature of this field. Bristol Myers Squibb was identified as the top productive institute, reinforcing the leading role of the United States in this area. Additionally, Wan XM was recognized as the most productive author. Analysing the global trend in this research area, the study identified the cost-effectiveness of PD-1/PD-L1 inhibitors and the comparative analysis of their economic impact in relation to other drugs as the prevailing hot topics. Thus, it is advisable for pharmaceutical companies involved in the development of novel PD-1/PD-L1 inhibitors to explore overseas markets to capitalize on this growing field. Finally, the study noted that developing countries engaged in health economics research on PD-1/PD-L1 inhibitors should prioritize the expansion of medical insurance coverage and expedite the marketing process of new drugs. These measures are essential in ensuring access to novel treatments and promoting advancements in healthcare systems.

Platelets, an essential component of blood responsible for clotting and stopping bleeding, have been found to display an important role in the invasion and spread of tumours. In a study conducted by Deng et al., the researchers conducted a comprehensive analysis of platelets in bladder cancer using data from multiple real-world cohorts. Their findings revealed two distinct patterns of platelet expression, named as Cluster 1 and Cluster 2, respectively. The first pattern, Cluster 1, was characterized with an inferior clinical outcome and showed a significant presence of cytokines, chemokines, and T-cell-related pathways. This suggests that Cluster 1 may represent a specific subgroup of bladder cancer with an immune-activated phenotype, making it potentially responsive to immune checkpoint inhibitor (ICI) therapy. To further support their findings, the researchers developed a robust risk score system known as the platelet risk score (PRS). This system consisted of 13 platelet-related genes and was validated using data from the GSE32894 and Xiangya cohorts. The PRS demonstrated satisfactory performance in predicting both prognosis and therapy response. Patients with bladder cancer divided into the high-risk group based on their PRS had a poorer prognosis but were found to be more sensitive to immunotherapy. This implies that patients in the high-risk group may benefit from targeted immunotherapy approaches. In conclusion, this study highlighted the significant impact of platelets on cancer progression and metastasis in bladder cancer. The identification of distinct platelet expression patterns and the development of the PRS provide valuable insights for predicting prognosis and guiding therapy decisions, particularly regarding the use of immune checkpoint inhibitors. Liposomal coenzyme A synthetase is an essential enzyme responsible for activating fatty acids and initiating the initial step of fatty acid metabolism. This metabolic process is categorized into four distinct groups, with medium-chain acyl-CoA synthetase (ACSM) being one of them. Interestingly, a study conducted by Li et al. indicated that ACSM6 might serve as a promising target for immunotherapy in bladder cancer. To gain deeper insights, they investigated the relationship between ACSM6 and the TME in BLCA. Astonishingly, their findings shed light on the role of ACSM6 in shaping the noninflammatory TME in BLCA while also providing the ability to predict the molecular subtypes of BLCA. Moreover, patients displaying low ACSM6 expression also showed a higher likelihood of responding positively to multiple therapies, including adjuvant therapy, neoadjuvant chemotherapy, and ERBB treatment.

Emerging evidence suggests a strong link between cell death and the development of tumors, as well as the response to ICI in various types of cancers. A recent study conducted by Li et al. aimed to construct a prognostic model for bladder cancer patients by examining genes related to both cuproptosis and ferroptosis (CFRGs). Utilizing a total of 5 CFRGs, the researchers constructed proportional hazards regression models. Remarkably, the high-risk groups identified in both the training and validation sets exhibited significantly poorer survival rates. Furthermore, the risk score was found to have a positive correlation with the tumor mutational burden (TMB), indicating its potential as a predictive marker. Conversely, the risk score showed an inverse relationship with tumor immune dysfunction and exclusion values, as well as tumor purity. Notably, the infiltration levels of antitumour immune cells and the expression of immune checkpoints were noticeably lower in the high-risk group. Additionally, the study observed a correlation between the risk scores and various pathway signals, including ErbB, MAPK, PI3K/AKT, mTOR, Hif-1 and TGF-β, suggesting their involvement in disease progression.

Immunogenic cell death (ICD) is an emerging mechanism of cellular death that triggers immune system activation and regulation against cancer. This unique process involves the release of various substances and antigens from deceased cells, which engage with antigen-presenting cells and other immune cells. In a study conducted by Su et al. applied several analytical techniques, to evaluate the prognostic significance of genes associated with ICD in patients with liver cancer. Through rigorous investigation, the researchers identified three genes, including PRNP, DNM1L and CASP8, as crucial prognostic markers for ICD. Subsequently, these genes were employed to construct a risk system that could categorize patients with liver cancer into high- and low-risk subgroups based on their ICD-related profile. To facilitate clinical application, a prognostic nomogram encompassing both the patients’ clinical characteristics and risk scores was devised, offering a comprehensive tool for personalized prognostication. In conclusion, this ground-breaking study highlights the importance of ICD-related genes and their potential for guiding patient management in liver cancer. The identified risk signature not only holds promise as a prognostic marker but also serves as a valuable resource for developing immunotherapeutic interventions targeting ICD in liver cancer patients.

In recent years, non-small cell lung cancer (NSCLC) has seen significant progress in immune and targeted therapies. However, the development of precision therapy has been hindered by the prevalence of cisplatin and ICI resistance in clinical settings. To address this issue, Li et al. conducted an in-depth analysis of public databases (GSE21656 and GSE108214) and identified protocadherin 7 (PCDH7) as a potential player in cisplatin resistance in lung cancer. They then conducted a series of *in vitro* experiments, confirming the oncogenic role of PCDH7 in NSCLC. Furthermore, the results of IC50 detection indicated a potential association between PCDH7 and cisplatin resistance in NSCLC. Interestingly, patients with high PCDH7 expression may exhibit increased sensitivity to bortezomib, docetaxel, and gemcitabine while showing resistance to immunotherapy. Finally, based on three genes correlated with PCDH7, the researchers developed a prognosis model that demonstrated a strong predictive ability for NSCLC patient survival. In conclusion, this study sheds light on the role of PCDH7 in cisplatin resistance in NSCLC and provides valuable insights into potential treatment strategies for patients with high PCDH7 expression. The findings also underscore the importance of personalized medicine in managing NSCLC and offer a promising prognostic model for predicting patient survival.

There has been a significant shift in the treatment strategy for NSCLC with the emergence of molecularly targeted therapies that focus on specific gene abnormalities. These targeted drugs have revolutionized the field; however, a major challenge remains in the form of tumor resistance to these therapies. To address this issue, Zhou et al. employed ESTIMATE algorithm to investigate the immune score. By analysing the immune score, the researchers were able to divide the patients into two distinct groups based on an optimal threshold. This division allowed them to identify differential genes that were associated with prognosis. Through a rigorous series of statistical analyses, they established a gene signature that could predict patient outcomes. To validate the accuracy of their findings, they conducted external validation using datasets GSE37745 and GSE31210, which further supported the prognostic model. These analyses revealed that pathways such as epithelial mesenchymal transition and immune-associated pathways were predominantly implicated in this group. Additionally, the researchers conducted somatic mutation and immune analyses to compare the differences between the two patient groups. This comparison provided valuable insights into potential drug sensitivity, which could serve as a basis for clinical treatment decisions. Further investigation led to the identification of two key prognostic genes: EREG and ADH1C. In summary, this research has shed light on the importance of the immune score in predicting survival outcomes for NSCLC patients. By identifying differentially expressed genes and understanding their involvement in key pathways, this study has provided valuable insights into potential treatments and prognostic markers for this challenging disease. With the continuous advancements in technology used for the detection of circulating tumor DNA (ctDNA), the importance of liquid biopsy as a valuable tool for prognostication and evaluating treatment response in patients diagnosed with NSCLC is increasingly acknowledged. A clinical trial conducted by Dong et al. included the participation of 90 individuals diagnosed with stage I-IIIA NSCLC. The aim of this trial was to evaluate the relationship between molecular residual disease (MRD) and various clinicopathological features, gene mutations, the tumor immune microenvironment, and treatment outcomes. Specifically, they discovered that the presence of ctDNA-MRD after surgery in NSCLC patients correlated with several factors, including primary tumor size, lymph node metastasis state, pathological subtype, presence of vascular invasion, and PD-L1 expression. Additionally, they observed that adjuvant EGFR-TKI targeted therapy was more effective than chemotherapy in eliminating ctDNA in postoperative patients with MRD.

TME plays a vital role in immunotherapy resistance, making it a significant factor to consider when examining lung cancer. Nanomedicine has emerged as a promising approach to enhance immunotherapy in this context. In Zhang et al. review, the authors shed light on the interplay between TME and immunotherapy, emphasizing the crucial role of TME in lung cancer immunotherapy (Zhang et al.). Additionally, they explored the potential of nanoparticles in regulating the TME to improve the effectiveness of immunotherapy. The authors also concluded that nanoparticle-based targeting of the TME could be a valuable strategy for solving resistance to PD-1/PD-L1 blockade in lung cancer.

NSCLC has emerged as a prime example of precision medicine, with the identification of multiple subtypes characterized by specific oncogenic driver mutations, leading to the development of targeted therapies at a molecular level. In recent years, significant strides have been made in the field of immunotherapy, particularly in the form of ICI, which involve the use of antagonistic antibodies to target the PD-L1-PD-1 axis for the treatment of NSCLC. In a study by Wang et al., the authors delved into the correlation between the types of immune cells within the TME and the effectiveness of immunotherapy in lung cancer. Additionally, they explored the efficacy of immunotherapy in the context of various gene mutations found in lung cancer, such as KRAS, TP53 and EGFR. Ultimately, the authors emphasize the potential of modulating immune cell populations within the TME as a promising strategy to enhance adaptive immune resistance in lung cancer.

Skin cutaneous melanoma, SKCM, known as one of the most lethal forms of cancer with a high likelihood of metastasis, poses considerable challenges for treatment. Current options such as chemotherapy, immunotherapy, and molecular therapy have not significantly improved the prognosis for SKCM patients, who face an alarmingly short median survival time. Recognizing the urgency of this issue, Sun et al. conducted an in-depth analysis of cuproptosis related signatures over-expressed in SKCM. Through their research, they developed an innovative risk stratification system that holds the potential to revolutionize the management of SKCM patients by enabling precise and targeted interventions. ROC values signify the robustness of the risk classification system in predicting the disease prognosis accurately. Furthermore, the study uncovered notable discrepancies between the low-risk and high-risk groups in terms of tumor burden mutational and immunology function, cell stemness characteristics, and drug sensitivity. These findings highlight the potential impact of the risk stratification system on tailoring personalized treatment plans for SKCM patients, ensuring optimum therapeutic outcomes. These findings further emphasize the potential of the risk stratification system in identifying gene expression patterns that correlate with disease progression and prognosis. In conclusion, the study conducted by Sun et al. sheds light on the pressing issue of SKCM and its poor prognosis. By thoroughly analysing cuproptosis-related differential genes, the researchers developed a novel risk stratification system that demonstrates efficacy in predicting disease outcomes. This system holds immense potential for guiding precise management and treatment decisions for SKCM patients, ultimately improving their overall prognosis.

Over the past decade, there has been significant research focused on unravelling the underlying mechanisms responsible for the development of acute myeloid leukaemia (AML). This increased attention has greatly enhanced our understanding of this disease. However, despite these advancements, the primary barriers to successful treatment remain resistance to chemotherapy and recurrence of the disease. Conventional cytotoxic chemotherapy often leads to acute and chronic side effects, making consolidation chemotherapy impractical, particularly in elderly patients. As a result, there has been a surge of interest in finding alternative approaches to address this issue. A comprehensive review conducted by Chen et al. has provided a thorough summary of various immunotherapies for the treatment of acute myeloid leukaemia. These therapies include immune checkpoint inhibitors such as CTLA-4 and PD-1/PD-L1, monoclonal antibody therapy targeting anti-CD33, anti-CD123, and anti-CD3/CD33 or CD3/CD123 bispecific antibodies. Presenting the recent progress in immunotherapy for AML, the authors also highlight the most effective therapies and major challenges in their implementation. In a related study, Li et al. conducted a comprehensive investigation into the clinical implications of genes involved in iron-associated cell death and apoptotic pathways in individuals with AML. The researchers developed a robust risk model based on four specific genes, which demonstrated promising prognostic value in both the training and validation cohorts. This model holds potential for aiding clinical decision-making and facilitating risk stratification in AML patients. To validate their findings, Western blot analysis was performed, indicating a significant decrease in the expression levels of C-Myc and Cyclin D1 after inhibiting CD4 expression levels. This result underscores the potential of iron-related cell death pathways as prognostic biomarkers and therapeutic targets in AML. Furthermore, this study emphasizes the importance of further exploration into the molecular mechanisms underlying iron balance, apoptosis regulation, and immune modulation within the bone marrow microenvironment. By shedding light on these interconnected processes, future research has the potential to significantly advance our understanding of AML and pave the way for innovative therapeutic approaches.

Liver metastasis from colorectal cancer is a significant risk factor that often leads to poor outcomes. Therefore, it is crucial to implement proactive interventions and treatments. Cancer-associated fibroblasts (CAFs) play a crucial role in the metastasis process, particularly through their involvement in metabolic reprogramming. However, the understanding of the relationship between CAF metabolic phenotypes and immune cells is currently limited. Wu et al. utilized both single-cell and bulk transcriptomics data to unravel the contributions of CAFs and immune cells in liver metastasis. They specifically focused on the metabolic subtype of CAFs and its impact. By decoding the roles of metabolism-related CAF subtypes and immune cells, the researchers developed a prognostic model that could effectively predict the outcomes of colorectal cancer patients with liver metastases. An intriguing finding of the study was the distinct interaction patterns observed between CAFs with different metabolic states and various immune cell types. Significantly, the researchers discovered that CAFs with varying metabolic profiles exhibited divergent communication patterns with different immune cell populations. This finding shed new light on the complex interplay between CAFs and immune cells during liver metastasis. Moreover, the study established that the prognostic features derived from CAF signature scores can serve as valuable indicators of the prognostic status of colorectal cancer patients. Samples with high CAF signature scores demonstrated elevated immune activity and an enrichment of tumor-related pathways. This suggests that a strong immune response and the activation of tumor-related pathways are associated with better outcomes in patients with liver metastases. Furthermore, the CAF signature score has shown practical utility in guiding the selection of chemotherapeutic agents with greater sensitivity. By considering this signature, clinicians can make more informed decisions regarding precision therapy for colorectal cancer with liver metastases. Therefore, this innovative approach has promising clinical implications for the management and treatment of such patients.

The discovery of AIR mechanisms and the development of therapeutic strategies to selectively inhibit AIR have proven to be effective in treating cancer patients, as demonstrated by the success of anti-PD therapy. Extensive efforts have been made to dissect, analyse, and classify the human tumour microenvironment (TME), leading to the identification of additional complex and diverse AIR mechanisms. However, it is pivotal to note that we have only just scratched the surface of this vast landscape. Nevertheless, it is possible to identify a few predominant or dominant AIR mechanisms that operate within the TME, which can guide the development of more targeted treatment approaches. By understanding and targeting these newly discovered AIR mechanisms, we can expect to achieve more efficient and effective treatment outcomes for a larger proportion of human cancers soon.

## References

[B1] Hegde P. S. Chen D. S. (2020). Top 10 challenges in cancer immunotherapy. Immunity 52, 17–35. 10.1016/j.immuni.2019.12.011 31940268

[B2] Morad G. Helmink B. A. Sharma P. Wargo J. A. (2021). Hallmarks of response, resistance, and toxicity to immune checkpoint blockade. Cell 184, 5309–5337. 10.1016/j.cell.2021.09.020 34624224 PMC8767569

[B3] Sharma P. Siddiqui B. A. Anandhan S. Yadav S. S. Subudhi S. K. Gao J. (2021). The next decade of immune checkpoint therapy. Cancer Discov. 11, 838–857. 10.1158/2159-8290.CD-20-1680 33811120

[B4] Vasan N. Baselga J. Hyman D. M. (2019). A view on drug resistance in cancer. Nat. Nov. 575, 299–309. 10.1038/s41586-019-1730-1 PMC8008476 31723286

